# Surface Topography Analysis of Mg-Based Composites with Different Nanoparticle Contents Disintegrated Using Abrasive Water Jet

**DOI:** 10.3390/ma14195471

**Published:** 2021-09-22

**Authors:** Kumari Bimla Mardi, Amit Rai Dixit, Alokesh Pramanik, Pavol Hvizdos, Ashis Mallick, Akash Nag, Sergej Hloch

**Affiliations:** 1 Department of Mechanical Engineering, Indian Institute of Technology (Indian School of Mines), Dhanbad 826004, India; kbmardi@gmail.com (K.B.M.); amitraidixit@iitism.ac.in (A.R.D.); mallick@iitism.ac.in (A.M.); akashnag1992@gmail.com (A.N.); 2 Department of Mechanical Engineering, Curtin University, Bentley, WA 6845, Australia; 3 Institute of Materials Research, Slovak Academy of Sciences, 04001 Kosice, Slovakia; phvizdos@saske.sk; 4 Faculty of Manufacturing Technologies, Technical University of Kosice, Bayerova 1, 08001 Prešov, Slovakia

**Keywords:** Mg-based nanocomposite, machinability, AWJ, surface topography, roughness, nanoindentation

## Abstract

This study investigated the effect of abrasive water jet kinematic parameters, such as jet traverse speed and water pressure, on the surface of magnesium-based metal matrix nanocomposites (Mg-MMNCs) reinforced with 50 nm (average particle size) Al_2_O_3_ particles at concentrations of 0.66 and 1.11 wt.%. The extent of grooving caused by abrasive particles and irregularities in the abrasive waterjet machined surface with respect to traverse speed (20, 40, 250 and 500 mm/min), abrasive flow rate (200 and 300 g/min) and water pressure (100 and 400 MPa) was investigated using surface topography measurements. The results helped to identify the mode of material disintegration during the process. The nanoindentation results show that material softening was decreased in nanocomposites with higher reinforcement content due to the presence of a sufficient amount of nanoparticles (1.11 wt.%), which protected the surface from damage. The values of selected surface roughness profile parameters—average roughness (Ra), maximum height of peak (Rp) and maximum depth of valleys (Rv)—reveal a comparatively smooth surface finish in composites reinforced with 1.11 wt.% at a traverse speed of 500 mm/min. Moreover, abrasive waterjet machining at high water pressure (400 MPa) produced better surface quality due to sufficient material removal and effective cleaning of debris from the machining zone as compared to a low water pressure (100 MPa), low traverse speed (5 mm/min) and low abrasive mass flow rate (200 g/min).

## 1. Introduction

To address certain challenges in manufacturing operations, which include machining high-strength and exotic materials to produce complex profiles with the required accuracy with respect to miniaturization, waste reduction and secondary operations, so-called unconventional technologies are used. The essence of these technologies is the use of energy (thermal, hydraulic, mechanical, chemical and electrical or a combination of these) rather than a solid cutting tool as used is in conventional machining. However, due to the introduction of heat to the material, which changes its microstructure, non-conventional thermal processes are not appropriate for some materials. During the disintegration/machining of materials with a water jet (WJ) or abrasive water jet (AWJ) [1], the temperature does not exceed 80 °C. This process leads to a lower concentration of stress in the material. The aforementioned temperature during disintegration and low dust levels make this process safe from the perspectives of potential explosion and environmental pollution. These features make WJ a universal technology based on natural processes, and the technology is now gaining importance due to global environmental problems [2]. With the exception of tempered glass, WJ is able to disintegrate all known types of materials, such as metals [3], non-metals, composites [4,5] and ceramics [6]. WJ is also used for flexible materials, such as rubber, polyurethane, foam rubber, carpets and any kind of sealing material, and it allows the mechanical disintegration of materials that emit carcinogenic, toxic or radioactive substances. The advantage of material disintegration technology is its versatility, as it is able to divide any material. Material loss ranges from 0.3 mm in the case of soft materials up to 3 mm when cutting hard materials. WJ is currently used mainly for the disintegration/machining of materials with special mechanical properties, including titanium alloys [7,8], difficult-to-machine alloys [9], rocks [10,11], glass [12,13], graphite [14] and different composite materials [15,16,17]. Technological parameters [18,19] have been studied in order to achieve the required surface quality in the disintegration of static [20] and/or rotating workpieces and to apply WJ and AWJ technology to the field of precision machining [21], especially difficult-to-machine and thermolabile materials [22,23,24]. Experimental analyses of topography and AWJ technology-related causes of roughness and waviness of the machined surface have been investigated in previous studies [25,26,27,28] in order to predict the surface roughness quality, which is also necessary for newly developed materials, including metal matrix nanocomposites (MMNCs).

MMCs reinforced with particulates are of exceptional importance, as they have a superior elastic modulus, higher strength-to-weight ratio, high-temperature resistance and near-isotropic and tailorable behavior [29]. However, MMCs reinforced with larger particles fail prematurely, as these reinforcements are susceptible to cracking during mechanical loading [30,31,32]. The increase in particle size may also reduce the tensile strength of MMCs when the reinforcement content is higher [33,34]. Decreasing the particulate size to the nanometer level has been observed to enhance the properties of MMCs. These are second-generation MMCs and called metal matrix nanocomposites (MMNCs).

There have been several primary investigations on the machining of MMNCs. Using a conventional machining process, magnesium-based MMNCs containing Ti and TiB_2_ particles of 50 nm were machined using a milling process [35]. Though Mg/TiB_2_ MMNCs exhibited better machinability, abrasion and chip adhesion significantly affected the cutting edges, while the reinforcement content and reinforcement materials affected the severity and type of wear. In addition, the depth of cut and speed had a considerable effect on the surface finish. A worse machined surface and a higher cutting force were observed at a lower feed (0.15–0.5 μm/tooth), revealing a stronger size effect. Micro-machining of Mg-MMNCs reinforced with 20 nm SiC particles (0, 5, 10, 15 vol.%) using a micro-milling process at various feeds and speeds was also studied [36,37]. The force during cutting increased with the increase in speed, feed and reinforcement content. A substantial increase in the slope was noted when the content increased from 5 to 10, which was in agreement with the significant variation in material properties with concentrations of around 5–10 vol.%. Nevertheless, the rate of increase in cutting force was much larger in the case of 60,000 rpm compared with 20,000 rpm and 40,000 rpm. In a non-conventional process, electrical discharge machining was applied to 1.5 wt% SiC-reinforced (50 nm) Al alloy using a copper tool electrode [38]. The pulse current was the prominent factor influencing all machining performance parameters, for example, electrode wear rate, material removal rate and surface finish. The optimized combination of variables was a pulse current of 8.00 A, voltage of 50.00 V, pulse-off time of 9.00 μs and pulse-on time of 8.00 μs. 

Magnesium metal matrix nanocomposites (Mg-MMNCs) demonstrate excellent mechanical properties that can be used for various applications, so it is essential to develop appropriate machining techniques, especially at the micro-scale, to promote their industrial application such as in the biomedical, automotive, chemical and aerospace industries [39,40]. However, machining is one of the major challenges in Mg-based metal matrix composites. Excessive tool wear develops in MMCs due to the presence of abrasive nanoparticles as reinforcements. Superfluous tool wear may cause excessive heat generation in the machining part during machining process. Pravir et al. [41] demonstrated a comprehensive study on the mechanical, thermal and wear behavior of Mg-nanocomposites. Furthermore, the traditional machining technique causes serious tool wear during the processing of MMNCs and results in higher surface roughness, which restricts the application of these composites. Sankaranarayanan et al. [42] have reviewed the research on the machinability of Mg-nanocomposites. Micromachining is one of the ways to avoid the above limitations. Gao and Jia [43] recently presented a finite element simulation to investigate the cutting force in micromachining of Mg-based nanocomposites. On the other hand, machining of magnesium-based materials without lubrication results in a greater risk of chip ignition, particularly smaller chips as they easily ignite when they come into contact with air at elevated temperatures and can compromise machining safety and output [44,45]. Therefore, non-traditional machining processes, such as laser-beam, electro-discharge, abrasive water jet and electrochemical machining, are also used for MMNCs [46]. AWJ machining or abrasive slurry jet machining is an important machining process in which pressurized water is used to apply suspended abrasive particles, for example, silicon carbide, aluminum oxide (Al_2_O_3_) or garnet. The material is removed by erosion without changing material properties due to the low heat generated in this process [47]. AWJ machining has been considered a key technology for processing micro features on hard-to-machine materials without applying excessive forces or causing thermal damage [48,49,50]. The machining performance of the AWJ depends upon various technological parameters or a combination of them. Some major input parameters are inlet water pressure, standoff distance, nozzle diameter, traverse speed, abrasive flow rate and type of abrasive grains used [51]. Water pressure determines the cutting capability and hydraulic energy of the jet, which is then transformed into kinetic energy exiting the nozzle [52]. Standoff distance determines the distance between the workpiece and the cutting head. Optimal level of standoff distance is required for effective cutting [53]. Nozzle diameter determines the volumetric flow rate of the water exiting the nozzle [54]. Traverse speed of the cutting head determines the distribution of the jet energy per unit length of the material and also determines the interaction time of the jet with the target material [55]. Abrasive flow rate determines the proportion of abrasive grains suspended in the water flow. Optimal setting of this parameter is required for better machining outputs [56].

It is clear from the above discussion that there have been limited studies on the effect of different amounts of nanoparticle reinforcements in magnesium-based MMNCs during water abrasive jet machining [57]. Conducting such studies is imperative, as micro-variation in the reinforcement content of nanocomposites significantly affects the properties of MMNCs. This study provides a detailed information on surface generation and surface integrity during AWJ machining when nano-reinforcement content in MMNCs varies. This paper investigates the AWJ machining of nanoparticle-reinforced MMNCs with different reinforcement content and different jet pressures, abrasive flow rates and traverse speeds. The results will provide a better understanding of the machinability of this material and improve the machining process, which will be beneficial to researchers and professionals in relevant areas.

## 2. Materials and Methods

The matrix alloy was made of 6% and 94% Al and Mg (99.9% pure), respectively, which were supplied by Alfa Aesar (Haverhill, MA, USA). Al_2_O_3_ nanoparticles approximately 50 nm in size were used as reinforcement and provided by Baikowski (Narashino, Japan). Al_2_O_3_ reinforcement particles at concentrations of 0.66% and 1.11% by weight were used to fabricate the MMNCs.

The disintegrated melt deposition (DMD) method was applied to fabricate the nanoparticle-reinforced MMNCs [58]. This is a hybrid casting process that combines elements of conventional casting and spray casting. This method involves adding reinforcement particles to the melt matrix by mechanical means. Afterwards, the melt slurry of the composite is disintegrated by jets of inert gas, which is normally oriented to the stream of the matrix melt. Subsequently, this melt is deposited onto a metallic substrate. The compositions of the MMNCs are listed in Table 1. The manufacturing of nanoparticle-reinforced MMNCs is still in at an early stage. The percentages of nanoparticles are generally maintained at around 2%, which results in reasonably good mechanical properties. The main objectives in manufacturing are to (a) maintain a uniform distribution of reinforced particles, (b) maintain the nano-size of the particles and (c) produce defect-free MMNCs. In this experiment, uniform properties of MMNCs were achieved with 0.66% and 1.11% weight with the existing facilities.

**Figure 1:**
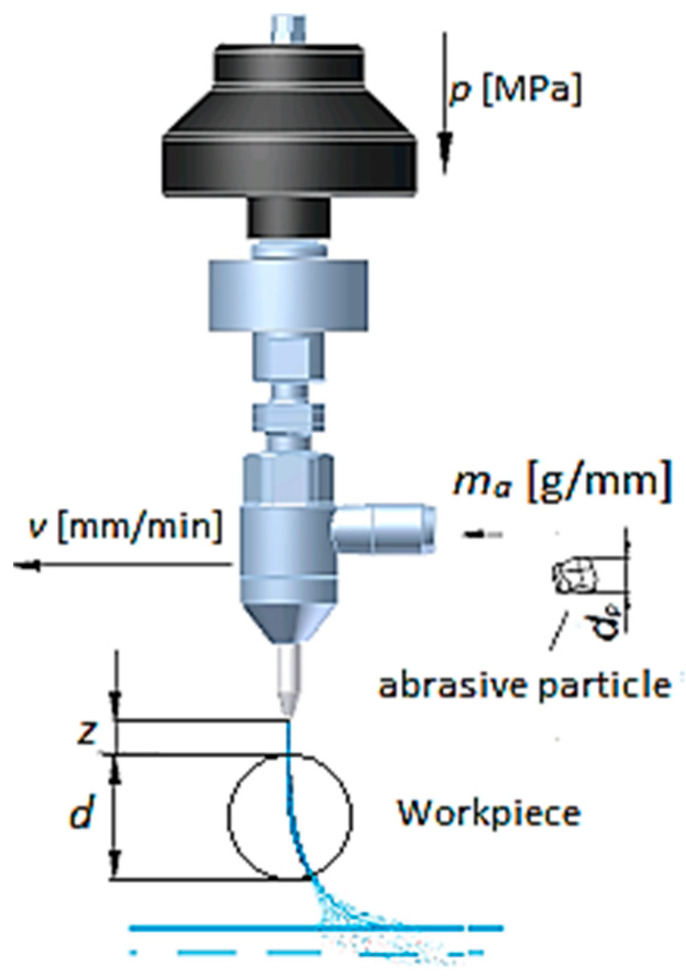
Diagram of cutting head with used parameters.

The AWJ machining process was performed on a PTV CNC WJ2020B-1Z-D machine. The machining variable limits were chosen based on a pilot experiment. The lower limits of the machining parameter combination, i.e., *p* = 100 MPa, *v* = 500 mm/min and *m_a_* = 200 g/min, were selected by determining the values at which cutting of the samples was achieved. The upper limit of supply pressure, i.e., *p* = 400 MPa, was limited by the maximum capacity of the hydraulic pump available during the experiments. Arbitrary values of traverse speed between the upper and lower limits were tested to observe the effect in this range. The experimental variables are displayed in Table 2. The parameter levels in each experiment are listed in Table 3. A detailed sketch of the cutting head of the abrasive water jet machine is illustrated in Figure 1. After machining, the 3D profiles of the machined surfaces were examined by a non-contact-type optical profilometer (MicroProf FRT, FRT GmbH, Bergisch Gladbach, Germany). The roughness of all surfaces was also obtained by the MicroProf FRT optical profilometer, in accordance with the EN ISO 4287 standard [59]. A sample area of 5 mm × 5 mm with 100 lines and 1000 points/line was selected for optical profilometry. During the scanning of the machined sample, the sample resolution was 50 µm and 5 µm in length and width, and the depth sensor had a vertical resolution of 1 µm. The morphology of the machined surfaces was further studied by FESEM.

Nanoindentation testing was carried out using the XP-Nanoindenter (Agilent, Santa Clara, CA, USA) before machining. After machining, the testing was performed on the Agilent G200 Nanoindenter in accordance with ISO 14,577 standard [60]. The indents were made in the cross-sectional plane below the machined surface of samples, and three rows of 10 indents were made at an increasing interval step of 5 µm down the machined surface. A load of 100 mN was applied to penetrate the surface to an average penetration depth of 2 µm. The mean and standard deviation of the hardness and elastic modulus values were obtained, and the results are discussed in Section 3.3.

## 3. Results

### 3.1. Surface Morphology

**Figure 2:**
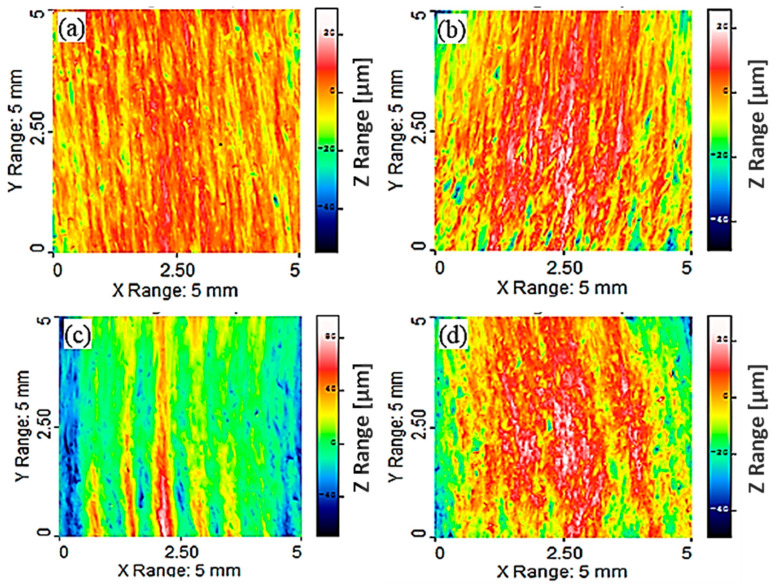
Surface topographical image of sample (**a**) A1; (**b**) A2; (**c**) A3; (**d**) A4 after experiment.

The profiles of the machined Mg-6Al/0.66 Al_2_O_3_ surfaces under different machining conditions are presented in Figure 2. Under all machining conditions, the surfaces are wavy, and the widths and heights of the peaks and valleys vary along the length. Some of these peaks and valleys are continuous, but a few are not. This is due to the uneven ploughing of the material by the abrasive grains at the interaction site of the material. It can be clearly observed that at higher traverse speed (*v* = 500 mm/min), the interaction time decreases, resulting in the generation of an uneven machined surface (z range = 166.3 µm). All peaks and valleys are inclined in the direction of the jet flow, though they are not parallel to each other in all cases. This inclination angle depends on the traverse speed of the jet and on material properties that resist the jet flow through the material. This is the result of the direction of the AWJ flow and the travel direction of the cutting head. Figure 2a shows the three-dimensional surface profile at a low traverse speed (*v* = 40 mm/s), where the maximum amplitude between the peak and valley is 84.15 µm. Some depressions on the machined surface are also observed. The distance between the height of the peaks and the valley depth decreases to 74.78 µm with the increase in traverse speed (*v* = 250 mm/min), though the size and number of depressions increase, as demonstrated in Figure 2b. With the further increase in traverse speed (Figure 2c), the distance increases significantly to 166.3 µm, where a larger part of the machined surface is depressed, and higher areas on the surfaces exist as thin discontinuous lines with varying thicknesses. This unevenness of the surface can be attributed to the shorter interaction time between the abrasive grains and the composite surface at higher traverse speed (*v* = 500 mm/min). However, at lower jet pressure (*p* = 100 MPa), abrasive rate (*m_a_* = 200 g/min) and traverse speed (*v* = 40 mm/min), the distance between the maximum peak height and valley depth becomes 78.17 µm, as illustrated in Figure 2d, which is due to the lower specific hydraulic energy transferred by AWJ to the composite sample, as determined by the combination of jet pressure, mass flow rate and traverse speed. This results in an even machined surface with a lower peak-to-valley distance (78.17 µm) as compared to other machining conditions.

**Figure 3:**
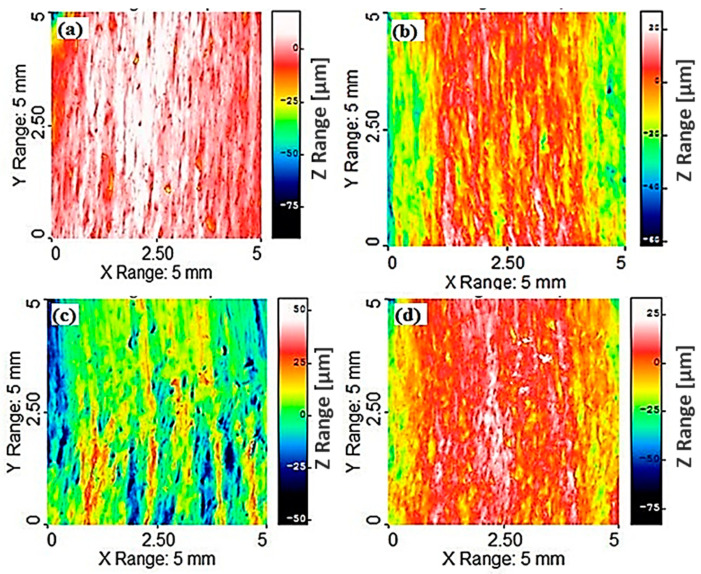
Surface topographical image of sample (**a**) B1; (**b**) B2; (**c**) B3; (**d**) B4 after experiment.

Machined surface profiles of Mg-6Al/1.11 Al_2_O_3_ under different machining conditions are shown in Figure 3. In these samples, surface depressions due to the impingement of abrasive particles are not observed at lower traverse speeds (*v* = 20 mm/min) (Figure 3a). This observation may be attributed to the enhanced mechanical properties of the fabricated composite due to the addition of 1.11% Al_2_O_3_ to the matrix material, which resists observable depressions in the machined surface at the current levels of machining parameters. As the traverse speed (*v* = 250 mm/min) increases, the peak-to-valley distance (88.13 µm) along the *Z*-axis decreases, though the depressions are more evident on the machined surface as compared to lower traverse speed, as presented in Figure 3b. The higher areas appear as a continuous line of different thicknesses, which is interrupted by depressions of different depths that form distinct grooves due to abrasive erosion. The maximum variation in the *Z*-axis becomes 108.0 µm with a further increase in traverse speed (*v* = 500 mm/min) (Figure 3c), and higher areas have the appearance of short lines of different thicknesses, which are interrupted and surrounded by depressions of different depths. The higher variation is due to the lower interaction time at the machining site between the abrasive and the composite surface. The variation in the Z range (117 µm) increases at reduced jet pressures (*p* = 100 MPa), abrasive rates (*m_a_* = 200 g/min) and traverse speeds (*v* = 40 m/min), which is due to the reduced hydraulic energy of the jet at the selected parameter levels, along with enhanced mechanical properties of the composite (1.11% Al_2_O_3_), which causes uneven machining of the sample (Figure 3d).

**Figure 4:**
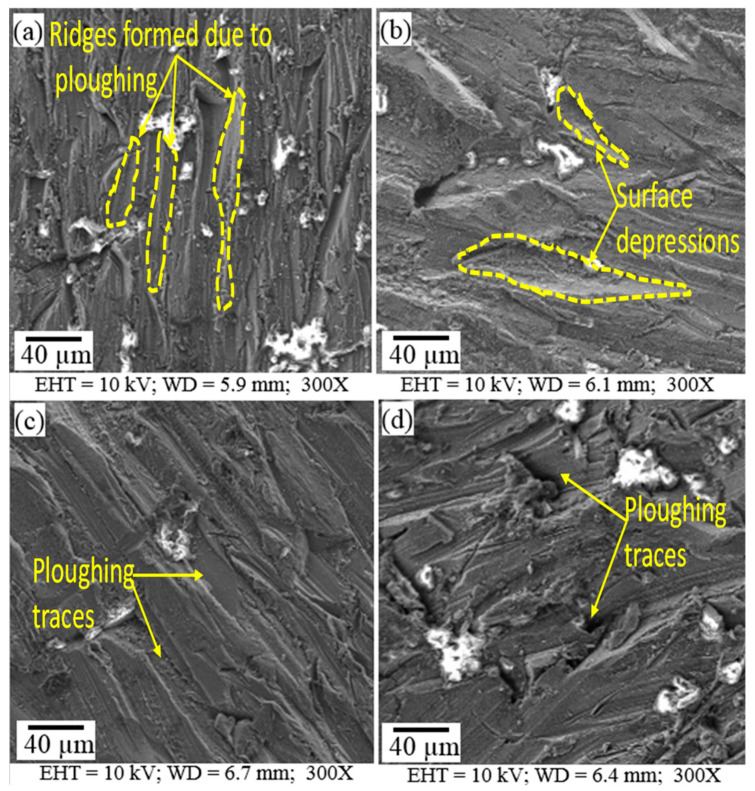
Surface morphological images of samples (**a**) A1; (**b**) A2; (**c**) A3; (**d**) A4 showing machined surface characteristics.

The machined surfaces of Mg-6Al/0.66 Al_2_O_3_ contain pronounced ploughing traces generated by abrasive grains, as shown in Figure 4. These traces are formed due to the interaction of the grains with the material. The grains plough the ductile material, leading to material disintegration. The ridges of the traces are very closely positioned and sharp when the traverse speed is lower (20 mm/min) (Figure 4a). At a slightly higher speed (250 mm/min), there are small oval depressions sparsely dispersed on the surface (Figure 4b). The ridges flatten and the distance between traces increases with the increase in traverse speed (500 mm/min) due to decreased interaction time between the abrasive grains and the material surface (Figure 4c). A very irregular surface with randomly oriented traces is generated when the traverse speed (40 mm/min), abrasive rate (200 g/min) and jet pressure (100 MPa) are low, as shown in Figure 4d.

**Figure 5:**
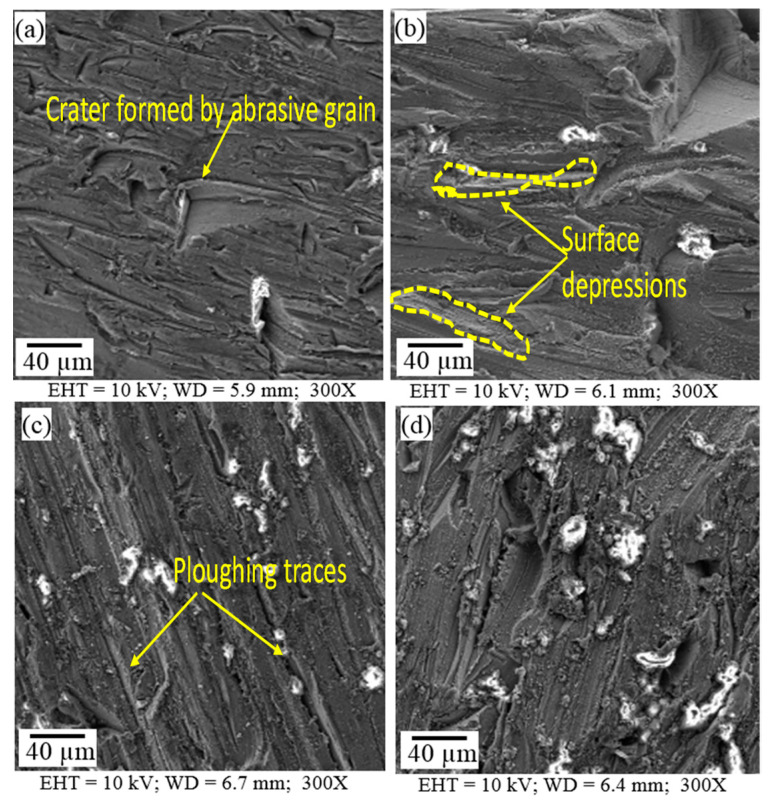
Surface morphological images of samples (**a**) B1; (**b**) B2; (**c**) B3; (**d**) B4 showing machined surface characteristics.

Traces of ploughing are also generated on the machined Mg-6Al/1.11 Al_2_O_3_ surface, as shown in Figure 5. In this case, the ridges of the ploughed traces are not very sharp, though they are located close to each other due to the higher concentration (1.11%) of Al_2_O_3_ reinforcement particles. This reinforcement concentration prevents the jet from deeply penetrating the material, generating blunt and closely packed traces. At lower traverse speed (20 mm/min), almost all traces are discontinuous, and very few continuous traces are present (Figure 5a). With the increase in traverse speed (250 mm/min), the traces coarsen, and the distance between them increases (Figure 5b). The traces sharpen and become straight, uniform and close to each other with the further increase in traverse speed (500 mm/min), as presented in Figure 5c.

**Figure 6:**
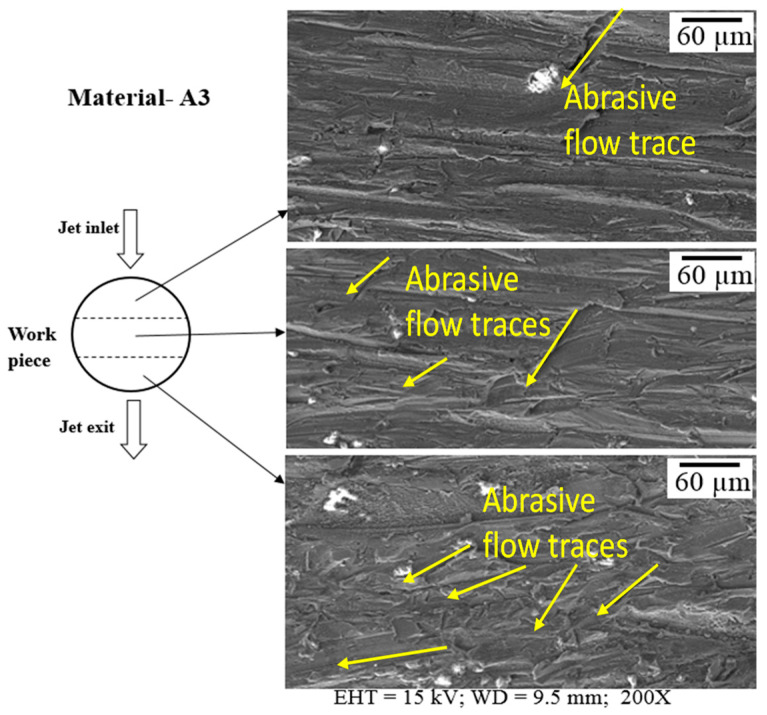
Surface along the depth of sample A3 after machining.

**Figure 7:**
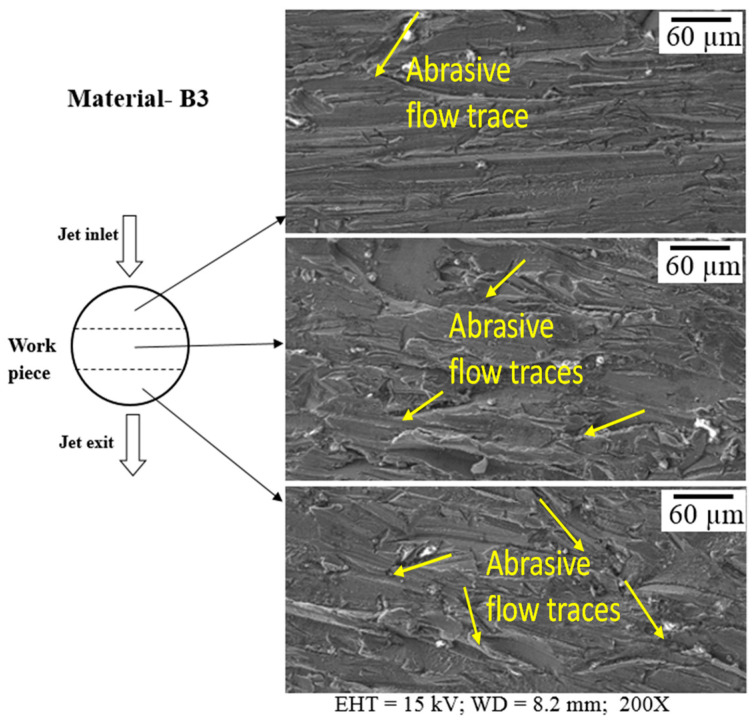
Surface along the depth of sample B3 after machining.

Variations in the surface along the depth of Mg-6Al/0.66 and Mg-6Al/1.11 Al_2_O_3_ samples are depicted in Figure 6 and Figure 7, respectively. The Mg-6Al/0.66 surface at the top of the specimen has numerous traces of abrasive flow, and many slightly curved longer and shorter lines in the direction of flow and a few medium-sized lines across the flow are evident. The longer lines are very distinct and spaced relatively far apart. The surface of the middle area of the sample is quite similar to that of the top area, but the longer lines are more densely packed. The surface of the bottom area of the sample is different to that of the top and middle areas. In this case, AWJ machining generates many randomly oriented distinct short lines on the surface. In addition, the bottom area of the machined surface, i.e., the jet exit region, appears to have many grooves and slots, which can be attributed to jet spreading or the non-uniformity of abrasive distribution in the jet.

The surface characteristics of Mg-6Al/1.11 Al_2_O_3_ are similar to those of Mg-6Al/0.66 Al_2_O_3_. In this case, the top region has many straight and curved lines, which are densely packed. The randomness of these curves on the surface increases towards the bottom of the sample. This is attributed to the loss of the hydraulic energy of the jet, which is necessary to overcome the resistance provided by the material with the increase in the width of the sample. Therefore, on the exit side, the surface roughness and randomness of the abrasive traces increase.

### 3.2. Surface Roughness

**Figure 8:**
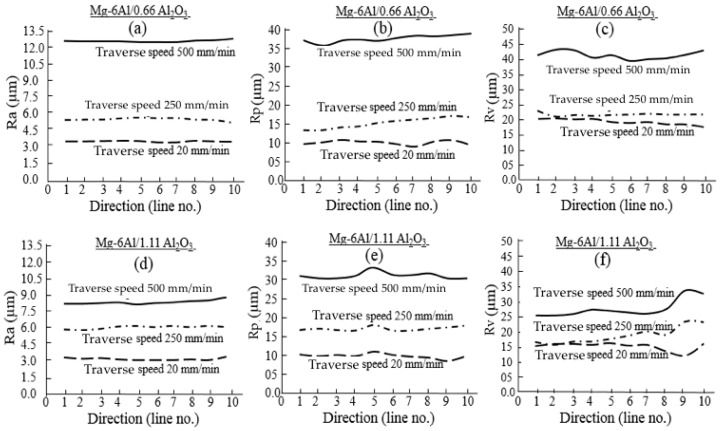
Effect of reinforcement content Mg-6Al/0.66 Al_2_O_3_ (**a**–**c**), Mg-6Al/1.11 Al_2_O_3_ (**d**–**f**) and traverse speed on surface roughness parameters Ra (**a**,**d**), Rp (**b**,**e**) and Rv (**c**,**f**) across the jet flow in different positions.

The roughness parameters Ra (average), Rp (maximum height of peak) and Rv (maximum depth of valley) were used to analyze the surface characteristics. These parameters were measured along 10 different parallel lines across the AWJ flow. Figure 8 presents the effect of varying the traverse speed on roughness at a constant abrasive flow rate. As expected, roughness is higher when higher traverse speed (500 mm/min) is used. This can be explained by the fact that fewer abrasive particles come into contact with the cutting surface at higher speed due to a shorter interaction time between the abrasive jet and the workpiece. Conversely, at lower speeds, more abrasive particles contact the cutting surface because of a longer interaction time between the abrasive jet and workpiece. Therefore, a smooth surface finish is produced. Figure 8a–f clearly shows a smoother surface at lower traverse speeds and a rougher surface at higher traverse speeds at the same abrasive flow rate. It is also evident that for both materials, the roughness values at 20 and 250 mm/min are almost the same. However, at higher speed (500 mm/min), Ra, Rp and Rv clearly differ. At higher traverse speed, metal matrix nanocomposites (MMNCs) with higher reinforcement content show lower roughness values than those of MMNCs with lower reinforcement content. This is due to the higher resistance of MMNCs (1.11% reinforcement), which protects the surface from the abrasive jet to produce irregular peaks and valleys. This is also demonstrated in Figure 4c and Figure 6c. It is believed that the roughness of MMNCs is mainly influenced by the micro effects of each impacting particle [61]. Since reinforcing particles (50 nm) in this composite are much smaller than the abrasive particle (177 µm), the nanoparticles will have little or no individual effect on the machined surface finish. However, the combined effects of nanoparticles influence the abrasive water jet machinability of MMNCs in this case.

Figure 8a,d show that the average surface roughness (Ra) does not vary notably with the directions of the measurements for either material at any traverse speed. For MMNCs with lower reinforcement content, the maximum height of the peak (Rp) gradually increases from direction L1 to L10 for traverse speeds of 250 and 500 mm/min (Figure 8b), but it is almost constant at low traverse speeds (20 mm/min), with one peak and one valley. When the reinforcement content is higher, a sudden peak in Rp is observed along direction line 5 at all speeds (Figure 8e). In addition, a valley is apparent for speeds of 20 and 500 mm/min along direction line 9. A peak of Rp indicates that the surface is very high; on the other hand, the valley of Rp indicates that there is a depression on the surface. All of these observations indicate the presence of transverse traces of particle flow crossing the direction of AWJ flow on the machined surface.

### 3.3. Mechanical Properties

**Figure 9:**
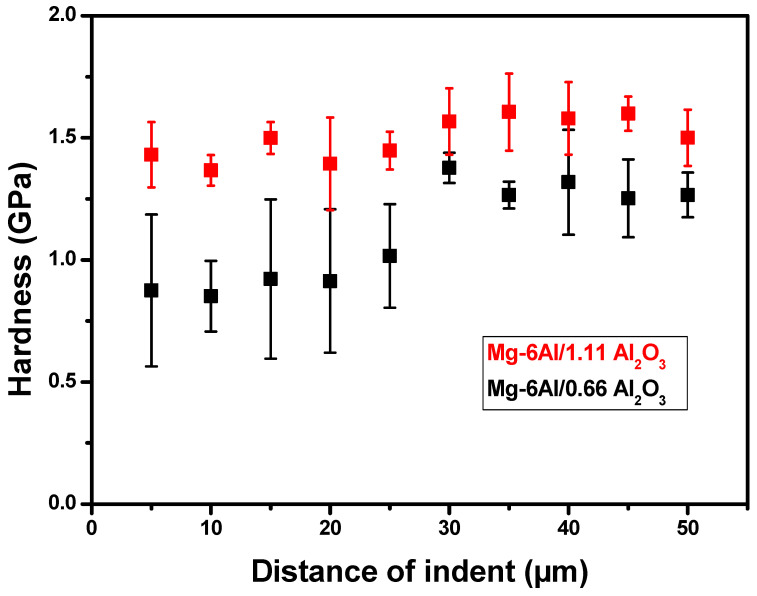

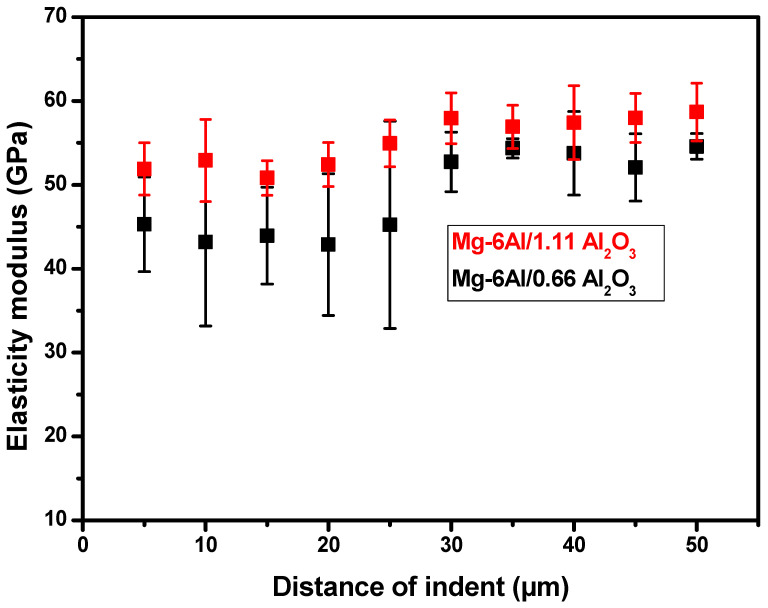
Variation in hardness and elastic modulus along the depth from the machined surface of both materials.

Changes in the hardness and elastic modulus along the depth from the machined surface for both materials are presented in Figure 9. The hardness of Mg-6Al/0.66 Al_2_O_3_ is reduced near the machined surface, and the deviation in the hardness is significant. The hardness also increases with the increase in depth and stabilizes at a lower deviation. The affected zone of Mg-6Al/0.66 Al_2_O_3_ appears to be at a depth of around 20–25 μm from the machined surface. The reduced hardness might be due to the softening of the material by AWJ machining. Moreover, near the machined surface, the deviation of the hardness can also be attributed to the peaks and valleys formed on the surface, which leads to the random orientation of grain boundaries, affecting the local hardness of the material. However, with the increase in reinforcement content, the hardness of Mg-6Al/1.11 Al_2_O_3_ does not change along the depth from the machined surface. Furthermore, the deviations of hardness values are significantly lower in this case compared to those of MMNCs with a lower concentration of reinforcement particles. Near the machined surface of Mg-6Al/0.66 Al_2_O_3_, the elastic modulus is low with a very high deviation, but it increases with the increase in depth, and the deviation decreases. On the contrary, the elastic modulus of Mg-6Al/1.11 Al_2_O_3_ is very steady without any noticeable variation along the depth from the machined surface. In this case, the deviation of values is very small. There is no doubt that this improvement in Mg-6Al/1.11 Al_2_O_3_ is due to the higher reinforcement content.

The hardness and elastic modulus values of Mg-6Al/0.66 Al_2_O_3_ range from 0.8 to 1.0 GPa and 42 to 45 GPa, respectively, up to a depth of 25 μm. These values are similar to the hardness and modulus values of unreinforced material. However, the values are consistently higher at depths greater than 25 µm. This might be due to the pull-out of reinforcements during AWJ machining since the reinforcement particles are much smaller than abrasive particles, and the number of particles is insufficient to simultaneously protect the surface from large impacting abrasive particles [55]. However, the increased particle content in Mg-6Al/1.11 Al_2_O_3_ is capable of protecting the machined surface from damage.

## 4. Discussion

Simultaneous erosion by the water jet and abrasive particles removes material during AWJ machining. Erosion by the water jet results from high-speed impingements of the jet or droplet (liquid streak) on the solid surface, where material is progressively removed and subsequently fails. Micro-cracking is the initial response of the target, which occurs due to microstructural irregularities, stress concentration around the slip steps and pre-existing flaws. Impacts of the water jet induce localized plastic deformation and a rough surface, which initiates micro-cracks in homogeneous bulk materials [62]. The water jet also generates cavitation erosion. The cavitation erosion process is described by cyclic deformation parameters [63], where the damage in material occurs through hydraulic penetration, stress wave propagation and lateral outflow jetting. The damage produced by these loading conditions on a material surface exposed to a single or multiple water drop impingements is responsible for the initiation of further damage and subsequent material removal [64]. Material removal by abrasive particles in ductile erosion occurs due to cutting and deformation processes, as in metal cutting or grinding. The impacting particle strikes the surface to develop an indentation and begins removing a chip of metal. The particle breaks due to impact, and fragments project radially from the primary site to develop secondary damage [65]. Due to repeated strikes by abrasive particles, deformation wear occurs on the target surface, which work-hardens the surface and initiates cracks. Propagation and distribution of the cracks result in material removal [66]. There have been investigations on target melting during erosion by abrasives, which have been related to the heating and melting of ductile materials subjected to erosive particles [67]. However, melting of magnesium alloy is unlikely because of its high thermal conductivity and the presence of high-speed water at room temperature, which is capable of removing heat from the erosion zone generated because of deformation in the target material.

In AWJ machining, the individual effects of the water jet and abrasive erosion complement each other. The water jet deforms the material and induces cavitation, which helps the particles to easily cut the material. On the contrary, the particles damage the surface by ploughing, indenting, embedding, work hardening and generating cracks, and then the high-speed water jet easily removes material from the damaged areas. In addition to reducing the temperature of the machining process, the water jet interacts with microstructural irregularities and defects and results in stress concentration, which produces tensile stress and initiates micro-cracking to remove materials [68]. 

The intensity of these different features depends on the machining conditions. At high water pressure (i.e., high jet speed) and low traverse speed, the surface features are more uniform (Figure 4a,b) compared to those obtained at a low water pressure, low traverse speed and low abrasive mass flow rate (Figure 4d) for the Mg-6Al/0.66 Al_2_O_3_ surface. This is because the weaker process parameters are able to damage the surface but are unable to clean it. However, smoother surfaces (Figure 4b,c) are generated at higher water jet pressure, traverse speed and abrasive mass flow rate. On the other hand, MMNCs with higher reinforcement content (Mg-6Al/1.11 Al_2_O_3_) have a greater ability to resist erosion. In this case, smoother surfaces are generated at lower traverse speeds (Figure 5a,b). With the increase in traverse speed, surface damage due to abrasive particles is reduced, and the effect of the water jet cavity increases, which is clearly visible in Figure 5c. With the reduction in water jet pressure and traverse speed, damage to the surface is primarily due to abrasive particles. Due to the ductile nature of Mg-6Al alloy, the embedment of abrasive particles is observed over the entire machined surface, irrespective of the machining conditions.

Most researchers have found that the centerline erosion rate caused by AWJ machining decreases with increasing stand-off distance. The reason behind this is that the radial expansion of the jet spreads, which reduces the number of strikes per unit area, though this does not significantly influence the velocity of particles [69]. However, a very short stand-off distance may impede abrasive flow from the tube. Significant variations in the abrasive flow rate are typical in the AWJ process, which is affected both by the spreading of the divergent jet with respect to the standoff distance and the depth of the workpiece, which affects the flow limit and size of the stagnation zone [70]. After the exit of slurry from the orifice, a slurry jet in air can be split into three distinct phases: (i) the starting phase, when the velocity in the potential core remains unchanged at its value at the exit of the orifice; (ii) the main phase in which the mean velocity of the flow decreases with distance from the orifice, and a surrounding mist phase arises; (iii) the diffused droplet phase, a comparatively low velocity phase included with the disintegration of jet into droplets [71,72]. In ASJM, strong deceleration of abrasive particles takes place due to the water stagnation zone near the target [49]. Erosion rate decreases with the increase in workpiece depth because of jet spreading during an increase in distance from the end of the effective nozzle to the bottom of the machined surface. It has also been reported that the central water jet splits up into droplets after an extended standoff distance depending on the water jet velocity as the jet entrains air with the abrasives in the upstream of the mixing tube [73,74]. Due to the spread of the jet, only a fraction of the original jet reaches higher depth and this fraction decreases with the increase in depth. In addition, particle velocity further decreases from drag within the stagnation zone close to the bottom of the channel [70].

The waviness of the machined Mg-6Al/0.66 Al_2_O_3_ surface was at its maximum (Figure 2c) at higher jet pressures, abrasive rates and transverse speeds. This might be due to lower resistance of MMNC to abrasion and high transverse speed when the abrasive jet does not get enough time to reduce the waviness of the machined surface. The maximum surface waviness of the MMNC with higher content of reinforcement (Mg-6Al/1.11 Al_2_O_3_) is lower, as shown in Figure 3. The highest waviness of the machined Mg-6Al/1.11 Al_2_O_3_ surface was noticed at lower jet pressures, abrasive rates and transverse speeds (Figure 3d). This uneven machining occurs due to the lower abrasive rate and jet pressure as this material has higher resistance to erosion.

The machined surface at the top is exposed to less diverged and greater amounts of abrasive jet compared to that at the bottom surface. This generates longer, straighter and sharper grooves at the top surface compared to that of the bottom surface for both materials. The higher reinforcement content of Mg-6Al/1.11 Al_2_O_3_ increases its erosion resistance, which reduces the groove length in the top surface compared to that of Mg-6Al/0.66 Al_2_O_3_.

## 5. Conclusions

The following conclusions can be drawn based on the analysis of the practicability of AWJ machining of MMNCs:

- The regular surface topography of the machined surface has been generated from AWJ machining at 20 mm/min and 250 mm/min traverse speeds for both material A (0.66%) and material B (1.11%), whereas at 500 mm/min traverse speed, the surface finish becomes rougher in material A compared to material B due to the lower resistance of the abrasive particles.
- Based on analysis of three-dimensional profiles it can be concluded that the depth of valleys and the size of depressions enhanced with traverse speed (20 mm/min–500 mm/min) whereas in material B at 20 mm/min traverse speed, no remarkable depressions were seen.
- The surfaces examined at three different regions with respect to jet inlet can be explained by the density of the striations in the AWJ machined surfaces, which increases from jet inlet to jet exit regions. The reason behind this fact is unsteady jet penetration process, non-uniformity of abrasive distribution in the jet and material resistance at the exit.
- The values of selected roughness parameters (Ra, Rp, Rv) increases from lower (20 mm/min) to higher traverse speed (500 mm/min) in the case of both material types. However, there is a large difference in roughness values for material A and material B at 500 mm/min speed. To some extent, better surface quality of material B can be achieved at higher speeds.
- The results from nanoindentation testing convey the softening of the AWJ machined surface up to the depth of 20–25 µm in the case of material A, whereas no significant variations in hardness, modulus values or softening phenomena were observed in material B.

## Figures and Tables

**Table 1:** Chemical composition of nanocomposites.

Composite (wt.%)	Mg-Al Alloy (wt.%)		Reinforcement	Reinforced Particles Size (nm)
	Magnesium	Aluminum	Al_2_O_3_ (wt.%)	
	94	6		
A: (Mg-6Al/0.66% Al_2_O_3_)	99.34		0.66	50
B: (Mg-6Al/1.11% Al_2_O_3_)	98.89		1.11	50

**Table 2:** Technological conditions of experiments.

Parameters	Symbols	Unit	Value
Water pressure	*p*	MPa	Variable 100, 400
Traverse speed	*v_t_*	mm/min	Variable 20, 40, 250, 500
Sample thickness	*h*	mm	8
Mass flow rate of abrasive	*m_a_*	g/min	Variable 200, 300
Abrasive size	*-*	Mesh (mm)	80 (0.177)
Diameter of nozzle	*d_o_*	mm	0.33
Diameter of focusing tube	*d_f_*	mm	0.9
Stand-off distance	*z*	mm	2
Position of cutting head	*φ*	°	90
Abrasive	*-*	-	Australian garnet

**Table 3:** Technological parameter levels used in each experiment.

Composite Materials	Experiment Number	Water Pressure (MPa)	Abrasive Flow Rate (g/min)	Traverse Speed (mm/min)
A: (Mg-6Al/0.66% Al_2_O_3_)	A1	400	300	20
	A2	400	300	250
	A3	400	300	500
	A4	100	200	40
B: (Mg-6Al/1.11% Al_2_O_3_)	B1	400	300	20
	B2	400	300	250
	B3	400	300	500
	B4	100	200	40

## Data Availability

The data presented in the study are available on request from the corresponding author.
